# Investigation of ZnO-decorated CNTs for UV Light Detection Applications

**DOI:** 10.3390/nano9081099

**Published:** 2019-07-31

**Authors:** Stefano Boscarino, Simona Filice, Antonella Sciuto, Sebania Libertino, Mario Scuderi, Clelia Galati, Silvia Scalese

**Affiliations:** 1 CNR-IMM, Ottava Strada n.5, I-95121 Catania, Italy; 2 STMicroelectronics, Stradale Primosole n. 5, I-95121 Catania, Italy

**Keywords:** MWCNTs, ZnO nanoparticles, nanocomposite layer, UV, room temperature, sensor

## Abstract

Multi-walled carbon nanotubes (CNTs) decorated with zinc oxide nanoparticles (ZnO NPs) were prepared in isopropanol solution by a simple, room-temperature process and characterized from structural, morphological, electronic, and optical points of view. A strong interaction between ZnO and CNTs is fully confirmed by all the characterization techniques. ZnO-CNTs nanocomposites, with different weight ratios, were deposited as a dense layer between two electrodes, in order to investigate the electrical behaviour. In particular, the electrical response of the nanocomposite layers to UV light irradiation was recorded for a fixed voltage: As the device is exposed to the UV lamp, a sharp current drop takes place and then an increase is observed as the irradiation is stopped. The effect can be explained by adsorption and desorption phenomena taking place on the ZnO nanoparticle surface under irradiation and by charge transfer between ZnO and CNTs, thanks to the strong interaction between the two nanomaterials. The nanocomposite material shows good sensitivity and fast response to UV irradiation. Room temperature and low-cost processes used for the device preparation combined with room temperature and low voltage operational conditions make this methodology very promising for large scale UV detectors applications.

## 1. Introduction

Zinc oxide has received great attention in several application fields due to its sensitivity to environmental factors such as humidity, gas species and light irradiation. Indeed, such factors can determine physico-chemical changes of ZnO that can be suitably used in photocatalytic [1,2,3] and sensing applications [4,5,6,7,8,9,10]. In the case of sensors where detection is performed by measuring electrical current changes taking place during exposure to gases, light irradiation, and so on, the high electrical resistivity of ZnO can be a hindrance. In these cases, it is often necessary to operate at high temperatures (200–500 °C) in order to measure a suitable current and get sufficient sensitivity. Much effort has been made to reduce the operating temperature to room temperature, mainly to reduce the energy waste during sensors operation and to avoid gas explosion in the presence of flammable and explosive gases. To this aim, several approaches [9] have been explored, like doping with metals, modifying ZnO surface/defectivity or, finally, preparing ZnO-based composites in order to increase the conductivity of the oxide [9,10,11,12,13,14,15,16]. However, most of the papers report the use of thermal treatments for the sensitive layer preparation, both for bare ZnO layers and for ZnO-based composites, and, furthermore, the use of relatively high (>150 °C) operating temperatures for sensing devices. Therefore, more effort still has to be concentrated on the study of sensitive materials that can operate as sensors at room temperature. Furthermore, for high-sensitivity sensors, the use of nanomaterials is mandatory, since a higher surface/volume ratio enhances the sensitivity to external factors (gas adsorption, light absorption, chemical reactions, etc.). Kwon et al. [17] reported the synthesis of ZnO-decorated CNTs for gas sensing applications, but their procedure involved sputtering (for catalyst deposition) and high-temperature thermal processes (500 °C) for the growth of ZnO nanoparticles. Another group [13] functionalized CNTs with ZnO by atomic layer deposition, using a temperature around 200 °C. It is clear that these preparation techniques cannot be performed on low-cost plastic substrates and this is not advantageous for large area industrial applications.

In this paper, we report on room-temperature, low-cost preparation of nanocomposite layers obtained by decorating multi-walled carbon nanotubes with zinc oxide nanoparticles and their applicability as sensitive layers for UV light detection at room temperature. The nanocomposites were characterized by different analysis techniques to investigate the morphological, structural, optical properties and, then, they were deposited at room temperature between two electrodes on a silicon dioxide/silicon substrate for electrical characterization under UV light irradiation in air. In particular, the oxide is the sensitive component of the system, and CNTs play the role of the electrical transducer. The advantages of the methodology described in this paper with respect to previous works are: room temperature deposition on localized regions of the device, no need for post-deposition thermal annealing processes, very low voltage and room temperature operation of the devices, and an ultra-small sensitive area.

## 2. Materials and Methods

### 2.1. Materials

Multiwalled carbon nanotubes (CNTs) with average diameter/length of 9.5 nm/1.5 μm, respectively, and carboxylic acid functionalization were purchased at Sigma-Aldrich S.r.l. (Milan, Italy). The –COOH functional groups present on carbon nanotubes allow to get a better dispersion in solution. A 2.5 wt. % dispersion of crystalline ZnO (hexagonal wurtzite) nanoparticles in isopropanol was supplied by Avantama AG (Stafa, Switzerland). The average diameter of the ZnO nanoparticles is about 15 nm.

### 2.2. Characterization

Absorbance spectroscopy was performed using a UV/Vis Cary 50 spectrophotometer by AGILENT (Santa Clara, CA, United States) in a wavelength range between 300 and 800 nm and Raman analysis was performed by a HORIBA (Kyoto, Japan) Scientific spectrometer (MFO model), using a He-Ne laser (632.8 nm wavelength). The spatial resolution was about 1 μm (micro-Raman technique).

The morphology and elemental composition of materials and devices was analyzed by a field emission scanning electron microscope (Supra35 FE-SEM by Zeiss, Oberkochen, Germany) equipped with energy dispersive X-ray (EDX) microanalysis system (X-MAX, 80 mm^2^ by Oxford Instruments, Abingdon, UK). Transmission electron microscopy (TEM) was used for a nanoscale structural characterization. To make the specimen suitable for TEM observation an isopropanol solution containing ZnO–CNTs nanocomposites was dropped out on a lacey-carbon TEM grid. TEM analysis was performed in a probe aberration-corrected JEM ARM200CF microscope by JEOL (Peabody, Massachusetts) at a primary beam energy of 60 keV, operated in both conventional TEM (CTEM) and in scanning TEM (STEM) modes. A GIF Quantum ER by Gatan Inc. (Pleasanton, CA, United States) was used for Electron Energy Loss Spectroscopy (EELS) measurements. More details are reported in the Supplementary Material.

For the electrical characterization of the materials we have used a Keithley 6430 Sub-Femtoamp Remote Source Meter (by Keithley Instruments—Tektronix Inc., Beaverton, OR, USA).

### 2.3. Samples and Devices Preparation

ZnO nanoparticles and CNTs were dispersed in isopropanol separately, using ultrasonic processes, and then the two solutions were mixed together, with different ZnO:CNTs weight ratios. CNT concentration was kept constant at 1 μg/mL for all the mixed ZnO–CNT solutions, and the ZnO concentration was varied (11 μg/mL, 4 μg/mL, 2 μg/mL, and 1 μg/mL), obtaining the samples named ZC11, ZC4, ZC2, and ZC1. The respective zinc oxide solutions (samples Z11, Z4, Z2, and Z1) without CNTs were prepared for comparison. All the solutions (names and description) are summarized in Table 1.

A silicon dioxide (SiO_2_) layer was thermally grown on a silicon substrate and two metallic electrodes (Pt 100 nm/Ti 20 nm) were defined by optical lithography and lift-off process [18]. The distance between the electrodes is 4 μm, and the width is 250 μm.

**Figure 1:**
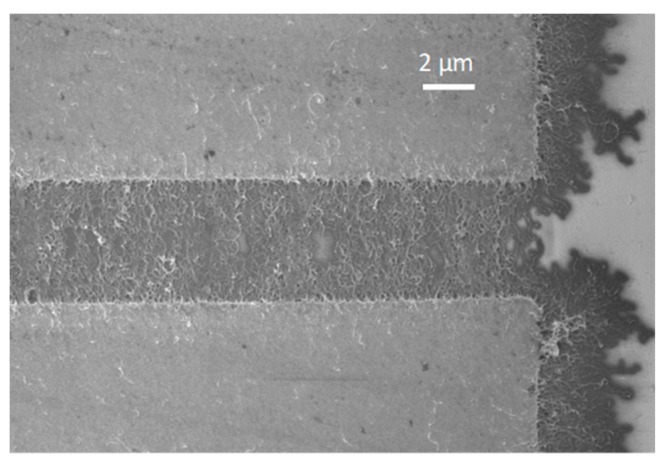
SEM image of a typical ZnO:CNT layer deposited between two electrodes for electrical characterization.

Different devices were obtained by depositing a nanocomposite sensitive layer from each solution between two electrodes in a 4 μm (distance between electrodes) × 250 μm (width of electrodes) region. The deposition of the nanomaterials was performed by dielectrophoresis, as described in a previous work [19]. A SEM image of a nanocomposite layer deposited between two electrodes is shown in Figure 1 as an example.

As a reference, a sample with a layer of CNTs was also prepared by the same procedure.

Besides the SEM analysis, EDX elemental mapping was performed on the devices in order to visualize the distribution of the different chemical elements on the prepared devices. An example is reported in Figure S1 of the Supplementary Material, showing the Zn and C signals due to the ZnO–CNTs layer deposited between the electrodes and the elements related to the substrate (Si, O) and the electrodes (Ti, Pt).

### 2.4. UV Irradiation

The UV irradiation was performed by the ASBN-D130 Deuterium Light Source by Spectral Products (Putnam, CT, USA), and different irradiance values of the lamp were used for the optical response measurements on the devices. The light source has been characterized by a fiber-optic spectrometer (AvaSpec-ULS2048L) as reported in Figure S2 of the Supplementary Material.

## 3. Results and Discussion

### 3.1. UV-Vis Absorption Analysis

**Figure 2:**
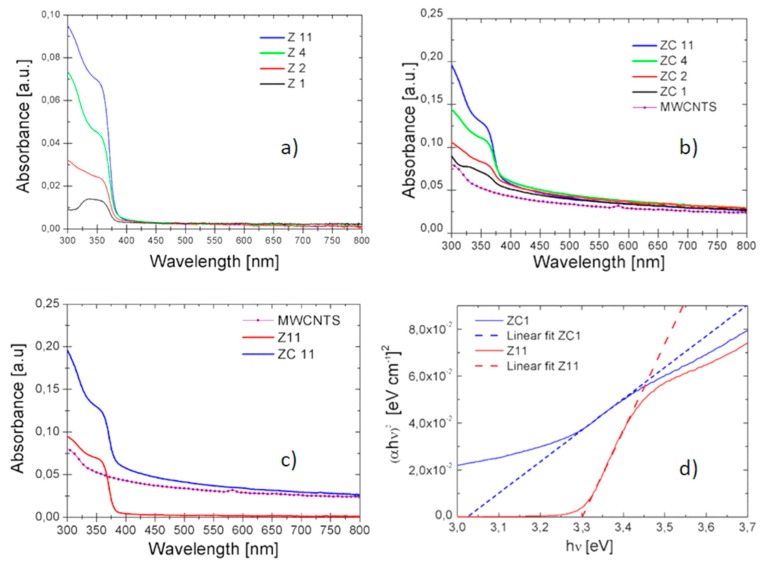
UV-Vis absorbance spectra of (**a**) ZnO solutions at different concentrations, (**b**) ZnO:CNTs at different weight ratios, but with the total ZnO concentrations comparable with the ones shown in (**a**); (**c**) a comparison among the spectra obtained for Z11, CNTs and ZC11 solutions; (**d**) Tauc plot for ZC1 and Z11 solutions.

The ZnO and ZnO–CNT solutions were analyzed by UV-Vis absorbance spectroscopy, and the spectra are shown in Figure 2. In particular, in Figure 2a–c we show, respectively, the absorbance spectra of (i) bare ZnO solutions at different concentrations, (ii) ZnO-CNTs mixed solutions at different weight ratios, with the total ZnO concentrations comparable with the ones shown in Figure 2a; (iii) a comparison among the spectra obtained for a CNTs solution, a ZnO solution (Z11) and the mixed ZC11 solution.

Figure 2a shows that the higher the ZnO concentration in the solution is, the higher the absorbance in the typical UV range (below 370 nm) is, as expected. A similar absorbance behaviour in the UV range is observed for the mixed solutions (Figure 2b), but a further contribution in the whole visible range of the mixed solutions is given by the presence of CNTs, as confirmed by the absorbance spectrum relative to CNTs (at the same concentration of 1 μg/mL), reported for comparison in the same figure. In order to evaluate the optical bandgap in the nanocomposite material, it is possible to use the Tauc method [20,21] and optical absorbance data can be plotted appropriately with respect to energy, that is (αhν)^1/*n*^ versus hν. In particular, h is Planck’s constant; ν is the photon’s frequency; and α is the absorption coefficient. The corresponding Tauc plots (for a direct allowed transition *n* = 1/2, as it is known for ZnO) are reported in Figure 2d. We plotted the spectra of Z11 (pure ZnO) and ZC1 (nanocomposite with the lowest ZnO:CNT weight ratio), since for a low ZnO:CNTs concentration ratio, we are confident that all (or most of) the ZnO NPs are interacting with CNTs, as we will show below in the electron microscopy analysis. The optical bandgap value for each solution is obtained by the intercept of the linear fit with the hν axis and we obtain that the nanocomposite material bandgap is about 0.24 eV lower than in the pure ZnO, suggesting an interaction between ZnO and CNTs. For solutions with larger ZnO:CNTs ratios, the difference with the pure ZnO optical bandgap becomes lower, and this can be ascribed to the presence of many ZnO particles not interacting with CNTs, showing the same optical absorbance of bare ZnO. As expected, for pristine ZnO (Figure 2a), no visible change of bandgap is observed as the concentration is varied.

### 3.2. Structural Characterization

**Figure 3:**
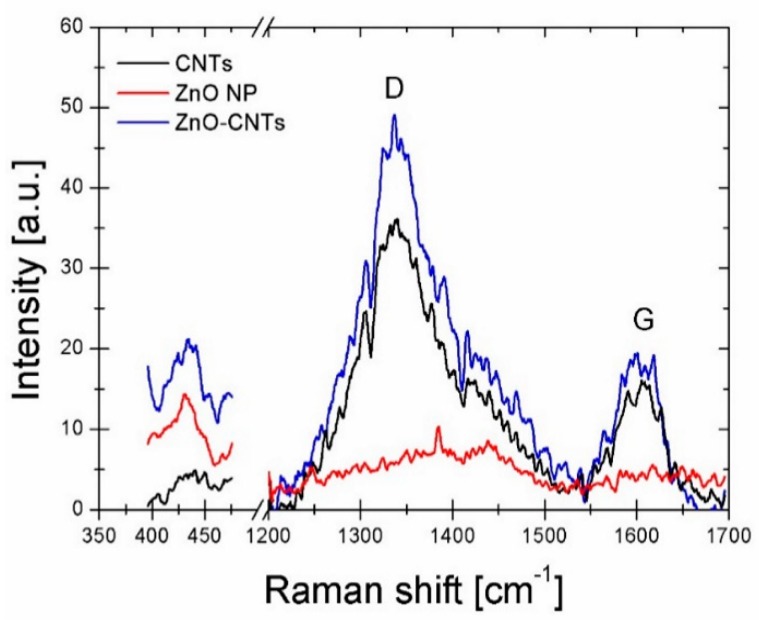
Raman spectra of CNTs, ZnO and a nanocomposite layer deposited on silicon substrates.

The three solutions reported in Figure 2c were dropped on silicon substrates and analyzed by Raman spectroscopy. The Raman spectra reported in Figure 3 show a peak at 435 cm^−1^ related to ZnO and the typical features related to carbon nanotubes in the region between 1200 cm^−1^ and 1700 cm^−1^. In particular, D and G bands are found at 1337 cm^−1^ and 1600 cm^−1^, respectively, and the I_D_/I_G_ ratio gives information about the disorder degree of the graphitic nanostructures, since D is related to sp^3^ hybridization (disorder) and G to the sp^2^ bonds (graphitic structure). By a fitting procedure, it is possible to measure the area of the D and G peaks and to calculate the I_D_/I_G_ ratio, that is 4.88 for CNTs and 5.33 for ZnO–CNT.

The increase of the I_D_/I_G_ ratio in the presence of ZnO confirms that the presence of the oxide nanoparticles induces disorder into the graphitic structure of CNTs. The small features between 1400 and 1450 cm^−1^ can be addressed to residual isopropanol contamination (CH_2_ bending vibration δ (CH_2_) and CH_3_ antisymmetric bending vibration δ_as_(CH_3_)) [22], resulting from the solution in which nanomaterials have been dispersed.

**Figure 4:**
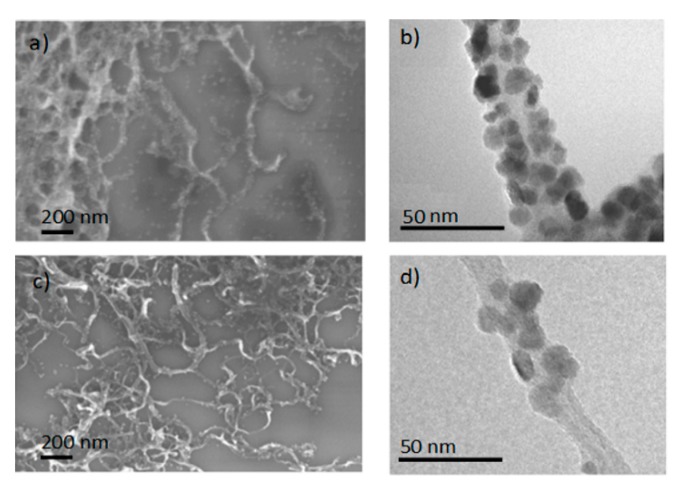
SEM (on the left) and TEM (on the right) images relative to ZC11 (**a**,**b**) and ZC2 (**c**,**d**) samples.

The morphology and the structure of the ZnO–CNTs nanocomposites were characterized by electron microscopy and the results relative to ZC11 and ZC2 are reported in Figure 4. SEM analysis of nanocomposite with larger ZnO:CNTs ratio (Figure 4a) shows the CNTs entirely decorated by ZnO NPs, and the presence of an excess of ZnO particles laying on the Si substrate and not anchored to CNTs. This does not occur on the sample with the low ZnO:CNT ratio (Figure 4c), indicating that the ZnO NPs bind to CNTs up to a complete covering and, afterwards, if more ZnO NPs are present, they remain dispersed in the solution. For low ZnO:CNTs ratios, it is possible that the ZnO NPs are not sufficient to decorate the CNTs entirely.

The SEM observations are also confirmed by the corresponding TEM micrographs showing the morphology of the ZnO–CNT nanocomposite. As a matter of fact, Figure 4b relative to sample ZC11 (higher ZnO concentration) shows how the CNT surface is completely covered by ZnO NPs, whereas the surface of sample ZC2 (lower ZnO concentration) is partially covered (Figure 4d). These images explain the effect observed on the absorbance spectra: Reducing the ZnO:CNTs ratio, all the metal oxide nanoparticles are in contact with CNTs resulting in a lower optical bandgap. On the contrary, increasing the ratio, an excess of free ZnO nanoparticles is observed, and the calculated optical bandgaps are closer to the value of the pure ZnO sample since they include both the contributions related to isolated ZnO nanoparticles and ZnO nanoparticles in contact with CNTs.

**Figure 5:**
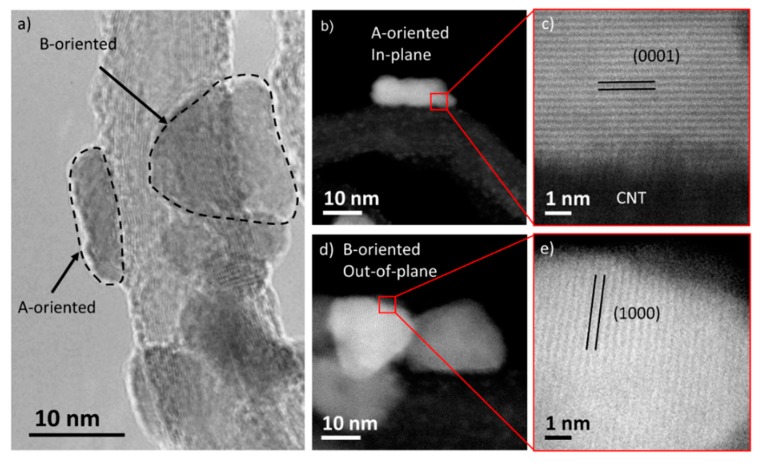
(**a**) TEM micrograph of sample ZC2. Dotted lines indicate two ZnO NPs with different orientations; (**b**,**d**) STEM micrographs of a ZnO NPs exposing an in-plane (**b**) and out-of-plane (**d**) view of 0001 planes; (**c**,**e**) High-resolution STEM micrographs relative to (**b**) and (**d**) respectively.

TEM and STEM analysis reveal that ZnO NPs possess a flattened disk shape and that the contact between the ZnO NPs and the CNT occurs mainly through the disk face, that is a 0001 plane. Indeed, on the TEM micrograph of Figure 5a, relative to sample ZC2, two ZnO NPs placed at the edge and at the centre of the CNT are marked as A- and B-oriented respectively (in-plane and out-of-plane view of the disk). Figure 5b shows an A-oriented NPs (in-plane view) with an elongated shape and the ZnO–CNT interface. Its high-resolution detail on Figure 5c shows that the interface is a (0001) plane. Figure 5d shows a B-oriented NPs (out-of-plane view) with a triangular shape. Its high-resolution detail on Figure 5e shows {1000} planes. This result is compatible with the orthogonality condition between A and B orientations.

**Figure 6:**
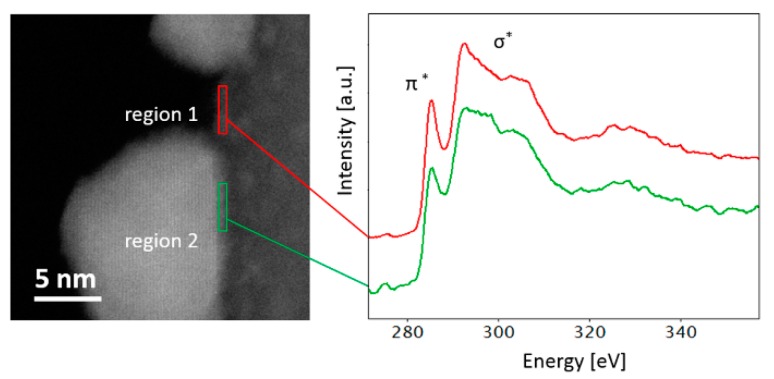
STEM image of the ZnO–CNT system and core EELS spectra of carbon K edge relative to the CNT outer surface (red) and at the CNT–ZnO interface (green).

In order to get a deeper understanding of the interaction between ZnO nanoparticles and CNTs and to investigate the nature of the bond between ZnO nanoparticles and CNTs, we have performed EELS spectroscopy at the ZnO–CNT interface. A careful analysis of EELS spectra can provide useful information about local atomic configuration [23,24]. In Figure 6, two high-loss EELS spectra of C K-edge acquired on the CNT edge (region 1) and at the CNT–ZnO interface (region 2) are shown. A fingerprint analysis shows that the shape of the σ* peak changes significantly on the two spectra.

In particular, the spectrum relative to region 1 shows a prominent and sharp 1s-σ* transition peak, consistent with the pure sp^2^ character of the CNTs structure, while the spectrum relative to region 2 shows a broadened peak. This can be hybridization, defects, etc., due to the contact (and interaction) between the CNT outer wall and the ZnO particle, compared to the perfect periodicity of CNTs, in agreement with Raman results.

### 3.3. Electrical Characterization & UV Sensing

The nanocomposite layers deposited between the electrodes, as described in Section 2.2, were characterized by I-V measurements, in dark conditions and under UV light irradiation. All the samples exhibit an ohmic behaviour, indicating that conductivity is mainly due to the CNTs, as shown in Figure S3 of Supplementary Material where the I–V curves obtained for ZC11, ZC4, ZC2, and ZC1 layers in dark conditions are reported. The samples were stored in air at room temperature and I–V measurements were repeated 2 months after the deposition (shelf life); they did not show any visible change, confirming high stability of the nanocomposite layers.

The samples were exposed to UV light irradiation in a wavelength range between 200 nm and 400 nm, for different irradiance values (see the spectral irradiance reported in Figure S2 of the Supplementary Material), and their UV response was analyzed. In particular, a voltage of 2 mV was kept constant between the two electrodes and the current flow through the deposited layers was measured with and without illumination and then converted to resistance. Before starting the measurements, all the samples were exposed to the UV irradiation for a “stabilization” time of about 1 h.

**Figure 7:**
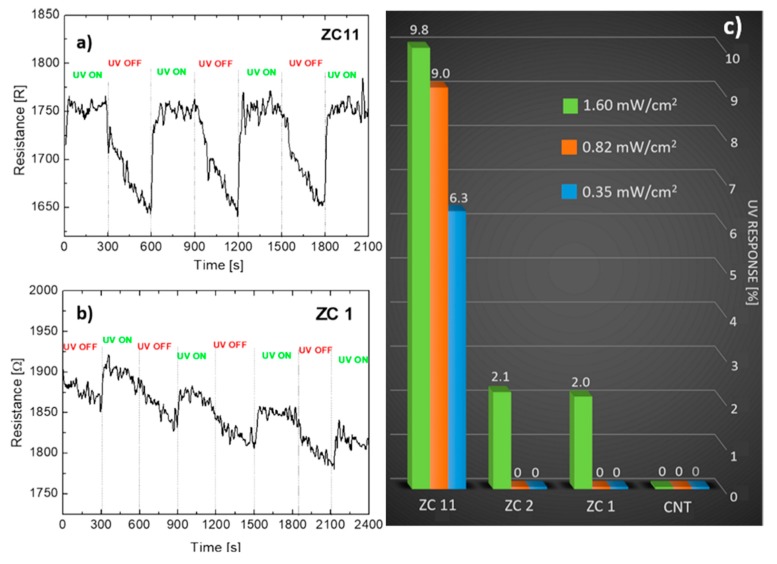
Electrical resistance measured switching on and off the UV irradiation on the (**a**) ZC11 (IrrUV = 0.35 mW/cm^2^) and (**b**) ZC1 nanocomposite layers (IrrUV = 1.6 mW/cm^2^) as a function of time. (**c**) Summary of all the responses obtained by devices with different ZnO:CNT ratios and for different irradiance values.

The UV lamp was switched on at least 10 min before opening the shutter and starting the electrical measurements under irradiation. We observed that ZC11 is sensitive to all the irradiance values used in this work, while ZC1 shows to be sensitive only for the maximum irradiance value (1.6 mW/cm^2^). The resistance measured (at V = 2 mV) as a function of time, when the UV irradiation is switched on and off, is reported in Figure 7a,b, for ZC11 at 0.35 mW/cm^2^ and for ZC1 at 1.6 mW/cm^2^ respectively. Under irradiation, the measured current flowing through the nanocomposite layer is stable and decreases as soon as the shutter between the lamp and the device is closed (UV OFF). The resistance, on the opposite, shows a clear and sharp increase each time UV light hits the device and a slower and more gradual decrease as the illumination is hindered by the shutter. This behaviour is perfectly reproducible as the shutter is opened and closed repeatedly (UV ON - UV OFF), as shown in Figure 7 for both the nanocomposites.

This holds for all the nanocomposites, and the higher the ZnO:CNT weight ratio is, the larger the current (resistance) change is, i.e., the electrical response. On the contrary, for the used irradiance values, the bare CNT layer does not show any significant sensitivity to UV illumination (no significant resistance change is observed passing from the dark to the illumination conditions and vice versa), as shown in Figure S4 of the Supplementary Material.

The response has been calculated for all the samples as the resistance difference between the UV ON and the UV OFF conditions (R_UVON_-R_UVOFF_) normalized by the resistance in the UV OFF state (R_UVOFF_). A summary of all the responses obtained by devices with different ZnO:CNT ratios and for different irradiance values are reported in Figure 7c. Our results show that the response of the nanocomposite material depends on the ZnO:CNT ratio and, in particular, the higher the ratio is, the higher the sensitivity to UV light is. Furthermore, for a constant ZnO:CNT ratio, we observe a different response as a function of the total irradiance, suggesting that these materials could provide not only qualitative information (ON–OFF condition), but also quantitative information about the source irradiance they are exposed to. Of course, this would require an optimization of the ZnO:CNT ratio and a calibration procedure for several UV irradiance values.

In order to describe and explain the observed behaviour, we have to take into account the possible reactions between the air molecules (mainly water and oxygen) and the sensitive ZnO–CNT layers with and without UV light. The ZnO–CNT sensitive layers, after deposition on the device, undergo a preparation process, that we call a “stabilization phase”, consisting of UV exposure for 1 h before operation. During this phase, the electrical resistivity of the nanocomposites reduces down to a plateau level, after that, the devices are ready for the operation. This plateau level is reached more quickly if the irradiation is performed under a nitrogen flux, suggesting that this process enhances the removal of molecular contaminants and humidity from the surface of ZnO particles. The behaviour shown in Figure 7 can be explained as follows: the UV irradiation induces the e^−^ − h^+^ pair formation, followed by O_2_ adsorption, according to the following reaction: O_2_ + (h^+^ + e^−^)→ O_2_^−^ + (h^+^). A charge transfer between ZnO and CNT occurs, determining a current decrease. Since the process is performed in air, water adsorption can also take place, probably just as physisorption.

**Figure 8:**
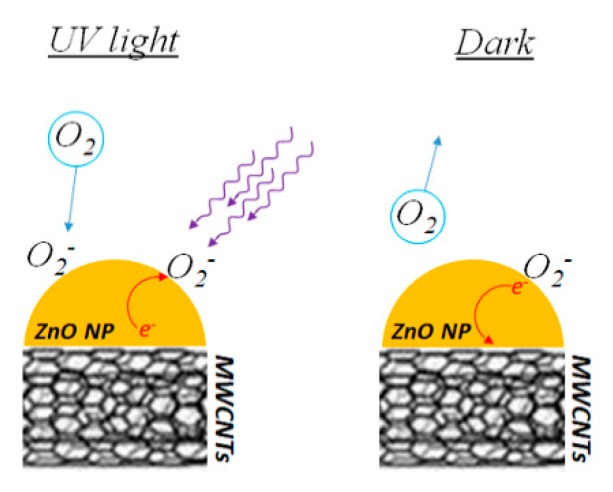
A scheme of the processes taking place on the ZnO–CNT system under UV light irradiation.

Indeed, water adsorption followed by molecular dissociation should induce a current increase according to the reaction: H_2_O + (h^+^ + e^−^) → 1/2 O_2_ + 2H^+^+2e^−^ + (e^−^). This is not observed, indicating that such a process is not favoured or is negligible with respect to the O_2_ adsorption. When the UV irradiation is switched off, the current increases again (no further e^−^ − h^+^ pair production), due to O_2_ desorption from the ZnO surface and release of electrons, flowing towards the CNT. A scheme of the processes is reported in Figure 8. Therefore, we may address all the resistance changes shown in Figure 7 as due to adsorption/desorption phenomena [25] and e^−^ − h^+^ pair formation (induced by UV irradiation) taking place on ZnO particles surface, in combination with charge transfer between ZnO nanoparticles and CNTs. CNTs are known to behave as electron acceptors and have been used in combination with semiconducting oxides (ZnO, TiO_2_, …) for photocatalytic and photoelectrochemical applications, in order to reduce electron-hole pair recombination and, therefore, to improve the photocatalytic properties of the oxides [26].

This charge transfer is possible due to the strong interaction between the two nanomaterials. In particular, the two nanostructures, by interacting in the solution, give rise to the ZnO–CNTs nanocomposite. The nanotube surface presents carboxyl groups which are able to interact with the oxygen of the ZnO and the interaction could be through hydrogen bonding, or the oxygen atoms of carboxyl groups interact with Zn atoms through the pair of electrons on the oxygen atoms [27]. By the characterization techniques (UV-Vis absorbance, Raman spectroscopy, SEM, TEM, EELS analysis) used in this study, we have clearly observed the effects of an interaction, that can be summarized as follows:

(i) Raman analysis shows an increase of the I_D_/I_G_ intensity ratio, that can be associated with a larger disorder on the graphitic structure. This means that, in some way, the presence of ZnO is affecting the sp^2^ graphitic order;
(ii) The optical bandgaps calculated for ZnO NPs and ZnO–CNT nanocomposites are different and, in particular, for the nanocomposite it is 0.24 eV lower than for the pure ZnO;
(iii) SEM-TEM analyses show the anchoring of ZnO NPs on the CNTs and the proportionality of ZnO coverage with the ZnO concentration in the solution, until the CNTs are entirely covered;
(iv) EELS analysis performed at the interface between the two nanostructures and at the free CNT surface shows a difference in the σ* peak of the C-K edge, indicating a change of the pure sp^2^ character of the CNT structure in contact with the ZnO NP, in agreement with Raman analysis;
(v) The electrical response of the ZnO–CNT nanocomposite under UV irradiation indicates that the light absorption by the ZnO NP affects the CNT conductivity, and therefore, a charge transfer occurs between the nanostructures.

The sensitivity, measured as the resistance change of the nanocomposite layer, depends on the ZnO coverage on the CNTs: the higher the coverage is, the more significant are the adsorption/desorption phenomena on ZnO and the charge transfer between ZnO and CNT.

## 4. Conclusions

In this work, we have reported a simple and low-cost methodology to prepare ZnO–CNTs nanocomposite layers, with different ZnO:CNT ratios. The nanocomposites have been characterized by morphological, structural, optical, and electrical techniques, confirming a strong interaction between the two components and showing fast electrical response to UV irradiation.

In particular, we observe that as the ZnO:CNT ratio is increased, the ZnO coverage of the CNT becomes complete. The interaction between the oxide particles and the nanotubes is confirmed by (i) a disorder increase of the CNT graphitic structure observed by Raman spectroscopy; (ii) a shift of the absorbance edge depending on the ZnO:CNT ratio shown by UV-Vis absorbance; (iii) a difference of the C-K edge features observed on the CNT and at the interface ZnO–CNT by EELS spectra; and (iv) the electrical response to UV light exposure.

The ZnO–CNT nanocomposite revealed to be very promising in UV-sensing application, due to the combination of optical and electrical properties of the two nanomaterials. In particular, our results suggest that an optimization of the weight ratio of the nanocomposite layer and a calibration procedure performed on the nanocomposite-based device could provide qualitative and quantitative information about the light source (ON–OFF condition and irradiance value). The main advantages of using such a nanocomposite material and our material/device preparation methodology can be summarized as follows: operation in normal environmental conditions (in air, at room temperature); minimum energy consumption due to very low operational voltage, i.e., 2 mV instead of typical 10 V [11,14,16] and no heating required; very small active area (10^–3^ mm^2^ instead of typical values between 0.1–1 cm^2^). The proposed methodology can be extended to low-cost substrates (flexible, plastic substrates) since it does not require high-temperature processes for sensor preparation nor high-temperature working conditions.

## Figures and Tables

**Table 1:** List of the solutions and corresponding ZnO concentrations or ZnO:CNTs concentration ratio used in this study.

ZnO Solutions	ZnO Concentration (μg/mL)	ZnO-CNTs Mixed Solutions	ZnO:CNTs Concentration (μg/mL) Ratio
Z11	11	ZC11	11:1
Z4	4	ZC4	4:1
Z2	2	ZC2	2:1
Z1	1	ZC1	1:1

## Supplementary Materials

The following are available online at https://www.mdpi.com/2079-4991/9/8/1099/s1, Section 1: EDX elemental mapping, Figure S1: Electron image of the inter-electrode region and the spatial distribution of the signals due to C (K_α_), Zn (L_α_), Si (K_α_), O (K_α_), Pt (M_α_), Ti (K_α_), Section 2: Experimental details about TEM and EELS, Section 3: Experimental details about UV irradiation, Figure S2: Spectra of the UV lamp used for the irradiation experiments, Figure S3: I–V curves obtained for different nanocomposite layers in dark condition, Figure S4: Resistance measured for a bare CNT layer during UV ON-OFF cycling at V = 2 mV.
