# Supplementary material for: Investigation of ZnO-decorated CNTs for UV Light Detection Applications
Source: Nanomaterials (Basel). 2019 Jul 31;9(8):1099. doi: 10.3390/nano9081099 (PMC6722801; doi:10.3390/nano9081099)
Supplement: Supplementary file 1 [file nanomaterials-09-01099-s001.pdf]

Supplementary Material

Investigation of ZnO-decorated CNTs for UV Light Detection Applications

Stefano Boscarino [1], Simona Filice [1], Antonella Sciuto [1], Sebania Libertino [1], Mario Scuderi [1], Clelia Galati [2] and Silvia Scalese [1,*]

[1] CNR-IMM, Ottava Strada n.5, I-95121 Catania, Italy
[2] STMicroelectronics, Stradale Primosole n. 5, I-95121 Catania, Italy
* Correspondence: silvia.scalese@imm.cnr.it

1. EDX elemental mapping

In Figure S1 the electron image and the element distribution of the inter-electrode region of a typical device is reported. The ZnO-CNT layer between the electrodes is clearly observed by the presence of Zn and C signal.

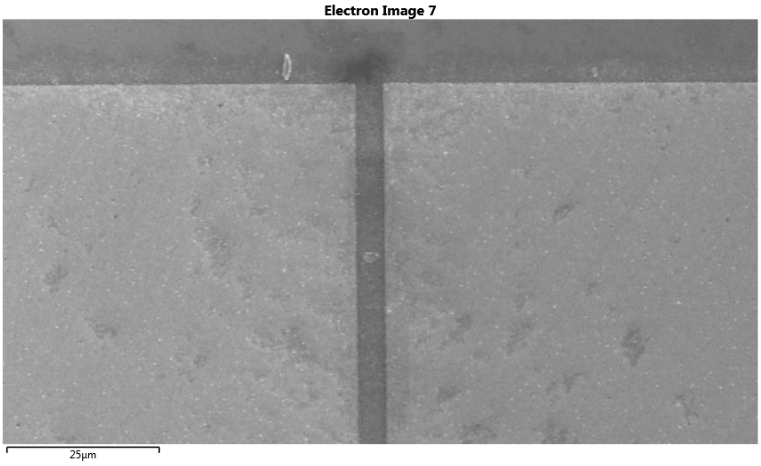

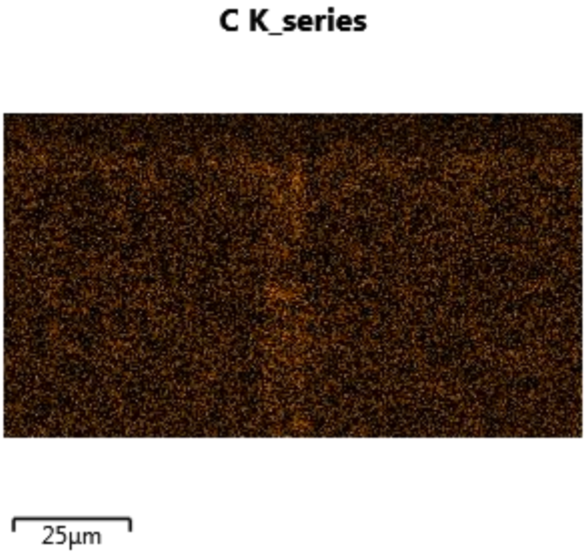

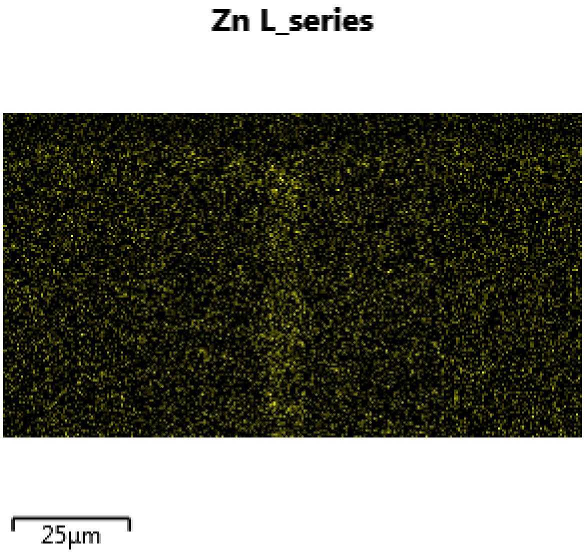

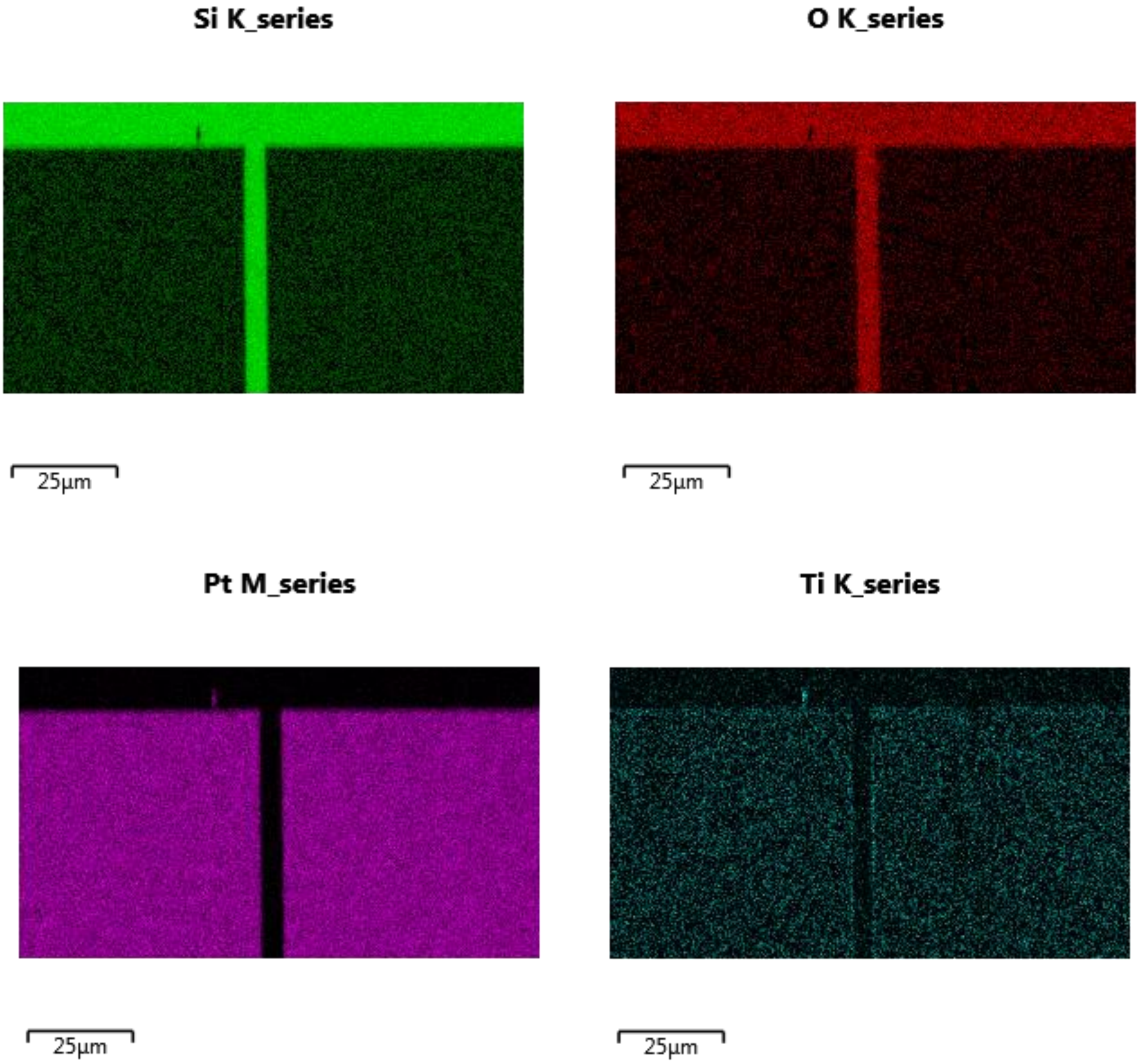

Figure S1. Electron image of the inter-electrode region and the spatial distribution of the signals due to C (K_{α}), Zn (L_{α}), Si (K_{α}), O (K_{α}), Pt (M_{α}), Ti (K_{α}).

2. TEM and EELS analysis

TEM analysis was performed in a probe aberration-corrected JEOL JEM ARM200CF microscope at a primary beam energy of 60 keV, operated in both conventional TEM (CTEM) and in scanning TEM (STEM) modes. The electron gun is a cold-field emission gun with an energy spread of 0.3 eV and the probe size was 1.0 Å. STEM micrographs were acquired in Z-contrast mode (High-Angle Annular Dark Field, HAADF).

A GIF Quantum ER was used for Electron Energy Loss Spectroscopy (EELS) measurements. Both low-and core-loss EELS spectra were acquired with the DualEELS capability through Gatan Digital Micrograph software. The use of Fourier logarithmic deconvolution on a full spectrum obtained by splicing together low-and core-loss EELS allows removing thickness-related plural scattering [1].

3. UV irradiation

The UV irradiation was performed by the Deuterium Light Source Spectral Products ASBN-D130. The spectrum of the lamp used for irradiation was acquired by the Fiber optic spectrometer AvaSpec-ULS2048L-USB2-FCPC (75 mm AvaBench, 2048 pixel CCD detector, USB/RS-232 interface) with grating UA - 300 lines/mm and bandwidth 200-1100 nm. The irradiance was varied, as shown in Fig.S2, and the calculated values are reported.

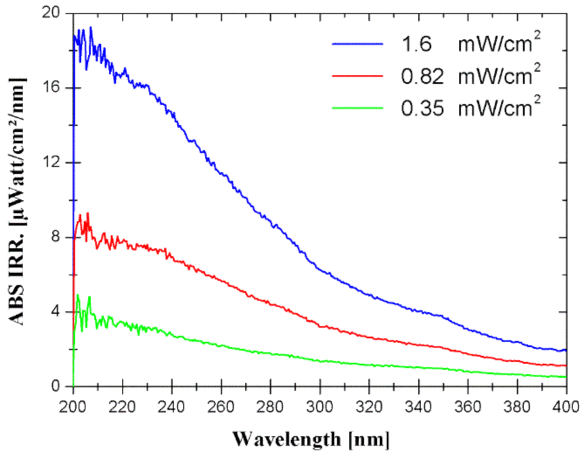

Figure S2. Spectra of the UV lamp used for the irradiation experiments.

4. I-V characteristics of ZnO-CNT layers

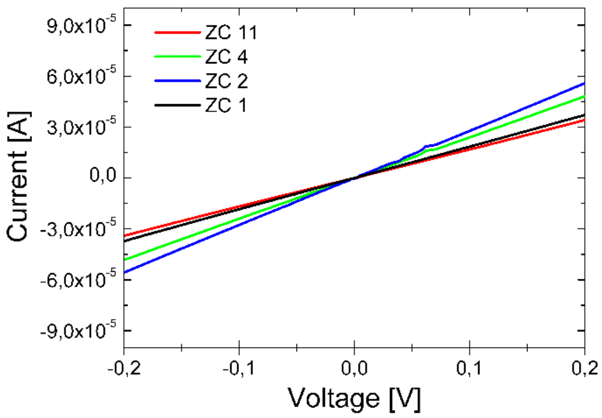

Figure S3. I-V curves obtained for different nanocomposite layers in dark condition.

5. Electrical behaviour of a bare CNT layer under UV illumination

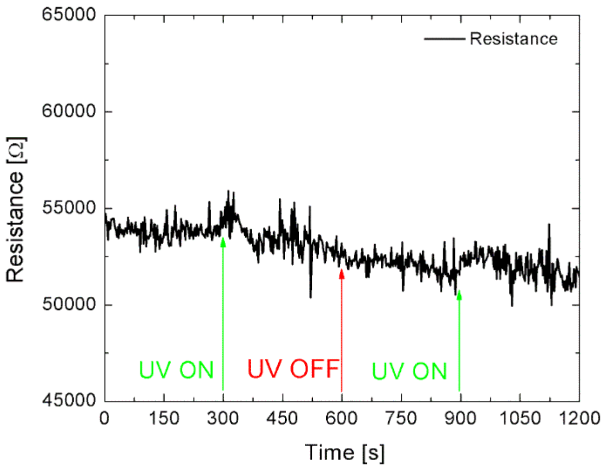

Figure S4. Resistance measured for a bare CNT layer during UV ON-OFF cycling at V=2mV.

Reference

[1] J. Scott, P.J. Thomas, M. MacKenzie, S. McFadzean, J. Wilbrink, A.J. Craven, W.A. Nicholson, Near-simultaneous dual energy range EELS spectrum imaging, *Ultramicroscopy*, 108 (2008) 1586. DOI: 10.1016/j.ultramic.2008.05.006.
